# Identification of Major Quantitative Trait Loci for Seed Oil Content in Soybeans by Combining Linkage and Genome-Wide Association Mapping

**DOI:** 10.3389/fpls.2017.01222

**Published:** 2017-07-12

**Authors:** Yongce Cao, Shuguang Li, Zili Wang, Fangguo Chang, Jiejie Kong, Junyi Gai, Tuanjie Zhao

**Affiliations:** National Center for Soybean Improvement, Key Laboratory of Biology and Genetics and Breeding for Soybean, Ministry of Agriculture, State Key Laboratory of Crop Genetics and Germplasm Enhancement, Nanjing Agricultural University Nanjing, China

**Keywords:** soybean, seed oil content, genome-wide association study (GWAS), linkage mapping, quantitative trait locus (QTL), single nucleotide polymorphism (SNP) markers

## Abstract

Soybean oil is the most widely produced vegetable oil in the world and its content in soybean seed is an important quality trait in breeding programs. More than 100 quantitative trait loci (QTLs) for soybean oil content have been identified. However, most of them are genotype specific and/or environment sensitive. Here, we used both a linkage and association mapping methodology to dissect the genetic basis of seed oil content of Chinese soybean cultivars in various environments in the Jiang-Huai River Valley. One recombinant inbred line (RIL) population (NJMN-RIL), with 104 lines developed from a cross between *M8108* and *NN1138-2*, was planted in five environments to investigate phenotypic data, and a new genetic map with 2,062 specific-locus amplified fragment markers was constructed to map oil content QTLs. A derived F_2_ population between *MN-5* (a line of NJMN-RIL) and *NN1138-2* was also developed to confirm one major QTL. A soybean breeding germplasm population (279 lines) was established to perform a genome-wide association study (GWAS) using 59,845 high-quality single nucleotide polymorphism markers. In the NJMN-RIL population, 8 QTLs were found that explained a range of phenotypic variance from 6.3 to 26.3% in certain planting environments. Among them, *qOil-5-1, qOil-10-1*, and *qOil-14-1* were detected in different environments, and *qOil-5-1* was further confirmed using the secondary F_2_ population. Three loci located on chromosomes 5 and 20 were detected in a 2-year long GWAS, and one locus that overlapped with *qOil-5-1* was found repeatedly and treated as the same locus. *qOil-5-1* was further localized to a linkage disequilibrium block region of approximately 440 kb. These results will not only increase our understanding of the genetic control of seed oil content in soybean, but will also be helpful in marker-assisted selection for breeding high seed oil content soybean and gene cloning to elucidate the mechanisms of seed oil content.

## Introduction

Soybean, *Glycine max* (L.) Merr, is an important grain and oil seed crop that is widely grown throughout the world, and provides more than 50% of the world’s oilseed production (Wilson, 2008; Soy Stats, 2011). As one of the determinants of oil production, soybean seed oil is an important trait targeted by many breeding programmers. Seed oil accounts for approximately 20% of the seed weight and it is a heritable quantitative trait in soybean. It is controlled by multiple quantitative trait loci (QTLs)/genes, which have small effects, and is also affected by the environment and its interaction with QTLs (Diers et al., 1992). Although seed oil content can be increased in traditional breeding based on phenotypic data selection (Burton and Brim, 1981), marker-assisted selection (MAS) is an efficient method that is independent of time and location (Allen, 1994; Chapman et al., 2003). Therefore, numerous studies have considered the genetics and QTL composition of seed oil content in soybean.

In the past 30 years, numerous QTLs for seed oil content have been identified in soybean (Diers et al., 1992; Lee et al., 1996; Orf et al., 1999; Csanadi et al., 2001; Chung et al., 2003; Hyten et al., 2004; Panthee et al., 2005; Pathan et al., 2013). However, most of these QTLs have low selection accuracy and have not been used effectively in MAS to breed for high seed oil content in soybean varieties (Collard and Mackill, 2008). It is also difficult to narrow down the QTL interval to the gene level based on a single-linkage mapping experiment. Especially in soybean, the size of the linkage mapping population is typically small. The main reason for this limitation is that the linkage mapping method has a relatively low genome resolution, which only uses the recombination events within the mapping populations (Darvasi et al., 1993; Beavis, 1994).

Genome-wide association (GWAS) is another method that takes advantage of a number of historic recombination events that have occurred within natural populations, and can overcome the limitations of linkage analysis (Rafalski, 2010). GWAS has been successfully applied to crop plants such as rice and maize (Zhao et al., 2011; Yang et al., 2014). In soybean, Mamidi et al. (2014) performed a GWAS to identify significant markers associated with iron deficiency chlorosis trait variations and identified seven major QTLs on seven chromosomes. Zhang et al. (2015) conducted a GWAS to dissect the genetic architecture of some agronomically important traits in early maturity soybean accessions, and detected 27, 6, 18, and 27 loci for days to flowering, days to maturity, duration of flowering-to-maturity, and plant height, respectively. Despite the fact that association studies have advantages in QTL mapping, the population structure (Q) and individual relationships may identify false positive correlations between the markers and the phenotype. Therefore, it is necessary to conduct linkage mapping in order to verify the results of a GWAS for dissecting complex traits.

Compared with traditional markers, single nucleotide polymorphisms (SNPs) have a more abundant DNA variation. Following the completion of the whole genome sequencing of soybean cv. Williams 82 (Schmutz et al., 2010) and the rapid development in sequencing technology, SNP markers are beginning to be used in soybean (Hyten et al., 2008). The next generation sequencing technologies enable researchers to quickly obtain numbers of SNPs throughout the genome, and have been successfully used in soybean for high-density genetic map construction and GWASs (Hwang et al., 2014; Song et al., 2016; Wang et al., 2016; Contreras-Soto et al., 2017). A specific length amplified fragment sequencing (SLAF-seq) technology with greater genotyping accuracy and relatively lower sequencing cost has been developed (Sun et al., 2013) and has been used to create high-density genetic maps in several plants, such as rice (Mao et al., 2015), soybean (Zhang D. et al., 2016; Cao et al., 2017), sesame (Zhang et al., 2013), and kiwifruit (Huang et al., 2013).

Seed oil content has a wide range of genetic variation among soybean accessions (Wilson, 2004) and there is also a strong negative correlation with seed protein content (Wilcox, 1998). Therefore, several QTLs can be related to both seed protein and oil content. Two major QTLs for seed oil/protein content have been mapped in many soybean populations: one was mapped at an interval from 20 to 40 centimorgan (cM) on chromosome (Chr.) 20 of the Soybean GmConsensus 4.0 Map and the other one was mapped at an interval from 10 to 30 cM on Chr. 15^1^. Many studies have focused on these two QTLs. The QTL on Chr. 20 was narrowed down to a small region (Nichols et al., 2006; Bolon et al., 2010; Hwang et al., 2014; Vaughn et al., 2014); and the QTL on Chr. 15 was fine mapped at a 535 kb interval (Kim et al., 2016). In contrast, fewer studies have focused on refining the position of the QTL for oil content on Chr. 05, which has been reported many times within a large genomic region (Mansur et al., 1996; Brummer et al., 1997; Orf et al., 1999; Pathan et al., 2013).

In the Jiang-Huai River Valley in China, soybean is grown from approximately June to October. However, little is known about the genetics of the seed oil content in these genotypes. The objectives of our study were to utilize the linkage and germplasm populations in various environments to map QTLs for seed oil content in soybean cultivars based on SNP markers, then dissect the genetic basis of seed oil content in these cultivars, and finally to refine a major QTL for seed oil content on Chr. 05 by combined linkage and GWA mapping.

## Materials and Methods

### Plant Materials and Plant Growth Conditions

For linkage mapping, two populations were used in this study. A recombinant inbred line (RIL) population was used for the initial QTL detection. The RIL population (NJMN-RIL) consisted of 104 F_2:8_ lines developed from a cross between *M8108* (a landrace material, seed oil content exceeding 20%) and *NN1138-2* (an elite cultivar characterized by high yield, seed oil content ∼ 19.0%). NJMN-RIL and its two parents were grown in five environments: Jiangpu Experimental Station, Nanjing, Jiangsu Province, in 2012 and 2014 (12JP and 14JP); Fengyang Experimental Station, Chuzhou, Anhui Province, in 2012 (12FY); Yancheng Experimental Station, Yangcheng, Jiangsu Province, in 2014 (14YC); and Huaian Experimental Station, Huaian, Jiangsu Province, in 2014 (14HA).

A secondary F_2_ population was used to confirm the most stable QTL from the initial mapping results. The F_2_ population consisted of 203 individuals developed from a backcross using *MN-5* with *NN1138-2*. *MN-5* was a line from the NJMN-RIL population with the *M8108* type–homozygous *qOil-5-1* region, while the *NN1138-2* type–homozygous was the other major QTL region identified in this study. The F_2_ population was grown at Jiangpu Experimental Station in 2015.

A total of 279 soybean accessions from the Yangtze-Huai soybean breeding germplasm population were used to perform the association study. The genotype of these soybean accessions can be obtained from our previous study (Li et al., 2016). The soybean accessions were grown in two environments: 13JP and 14JP.

The NJMN-RIL and germplasm populations were grown in a randomized complete block design, with three replications from approximately June to October. A 1 m single row plot of 10 plants per RIL/accession was grown, with a distance of 10 cm between plants and a row spacing of 50 cm. The secondary F_2_ population was grown in rows with a length of 2 m, with 50 cm between rows and 20 cm between plants. Field management was performed under normal conditions.

### Measurement and Analysis of Phenotypic Data

Seed oil content was measured by an Infratec^TM^1241 near infrared analysis (NIR) Grain Analyzer (Foss, Hillerød, Denmark) on a 10% moisture basis using approximately 15–20 g samples. Seed oil content for each RIL and soybean accession was an average of three replications within a planting environment.

The frequency distribution of phenotypic data was calculated using the SPSS Statistics 20.0 software (SPSS Inc., Chicago, IL, United States). The broad-sense heritability (*h*^2^) and analysis of variance (ANOVA) tests were conducted using the SAS PROC generalized linear model (GLM) program. The *h*^2^ of seed oil content in RIL and germplasm population was estimated using the following equation:

$$h^{2}\;=\;\sigma_{g}^{2}/(\sigma_{g}^{2}+\sigma_{ge}^{2}/n+\sigma_{e}^{2}/nr)$$

where *σ*^2^_g_ is the genotypic variance, σ^2^_ge_ is the variance of the genotype-by-environment interaction, σ^2^_e_ is the error variance, *n* is the number of environments, and *r* is the number of replications within an environment (Nyquist and Baker, 1991).

### Genotyping

For the NJMN-RIL population, approximately 1 g of fresh leaves obtained from two parents and individuals of each RIL were used to extract the genomic DNA using the cetyltrimethylammonium bromide method (Doyle, 1990). All RIL individuals and the two parents were genotyped using SLAF-seq technology to generate genome-wide SLAF markers. SLAF library construction, high-throughput sequencing, and high-quality SLAF marker acquisition were performed as described by Sun et al. (2013). The soybean reference genome (*G. max* Wm82.a1) was used to determine the physical position of the high-quality SLAF markers.

For the secondary F_2_ population, a total of 31 pairs of simple sequence repeat (SSR) markers based on the soybean SSR database (Song et al., 2010) and four pairs of *InDel* markers based on the results of Lam et al. (2010) and distributed across the *qOil-5-1* region (38–40.5 Mb on the Chr. 05), were used to screen for polymorphisms between *MN-5* and *NN1138-2*. Finally, 10 polymorphic pairs (one *InDel* marker and nine SSR markers) were used to genotype the F_2_ population and then construct a genetic map covering the *qOil-5-1* region.

A set of 279 soybean germplasm lines was assembled for GWAS. All lines were genotyped for SNP markers using high-throughput genotyping platforms, as described by Li et al. (2016). Finally, 59,845 SNPs, with minor allele frequencies (MAF) > 0.05, were available for the GWAS.

### Construction of Linkage Maps for the NJMN-RIL Population and QTL Linkage Mapping

After genotyping the 104 RILs, all polymorphic SLAF markers were filtered four times and further quality assessed as described by Sun et al. (2013). A SLAF with less than three SNPs and an average depth for each sample above three was considered a high-quality SLAF marker. Then, all high-quality SLAF markers were used for construction of the linkage map. The linkage map was constructed using High Map software (Liu et al., 2014). The markers were divided into linkage groups (LGs) using the single-linkage clustering algorithm at a logarithm of odds (LOD) threshold ≥3.0. The Kosambi mapping function (Kosambi, 1943) was used to calculate the map distances in cM from the recombination frequencies.

The QTLs were detected using WinQTLCart 2.5 software. The window size, the working speed, and the control marker number were set at 10 cM, 1 cM and 5, respectively. Model 6 (standard model) for composite interval mapping was used to identify QTLs in each environment, with a log likelihood of 2.5 set as the threshold of an existing QTL (Wang et al., 2007).

### The Q and Linkage Disequilibrium (LD) of the Soybean Breeding Germplasm Population

The Q and the kinship matrix (K) for these 279 soybean accessions were calculated as previously described (Li et al., 2016). The 279 soybean materials could be divided into three major subpopulations. The LD was approximately 480 kb, whereas *r*^2^ dropped to half its maximum value (Li et al., 2016). The LD blocks were within the interval of a QTL based on an LD analysis using the *r*^2^ correlation between each marker using the HAPLOVIEW 4.2 software (Barrett et al., 2005). The parameters in the program included MAF (≥0.05) and the integrity of each SNP (≥50%).

### Genome-Wide Association Mapping

In this study, the GWAS was performed using the TASSEL 5.0 software (Bradbury et al., 2007). Two statistical models were considered: (1) the GLM model without the Q and K; and (2) the mixed linear model (MLM) with the Q and K, which regarded Q and K as fixed and random effects. Compared with the GLM method, the MLM model can reduce the number of false positive results and was considered more appropriate for this study. To obtain more reliable results, the Bonferroni threshold (*P* < 1/59845, -log_10_*P* > 4.78) was used as the threshold to identify significant association SNPs. The association region was also estimated by extending 480 kb (LD distance) upstream and downstream of the most significantly associated SNP position.

## Results

### Phenotypic Evaluation of Seed Oil Content

The phenotypic performance is presented in **Table 1** and Supplementary Figure S1. For the NJMN-RIL population, in the female parent, *M8108* had an average seed oil content of ∼20.5%, and *NN1138-2* of ∼19%, with these differences being significant in all environments (*P* < 0.001). A continuous distribution and transgressive segregation were observed for the trait in the RIL population in all environments. Due to the kurtosis and skewness (absolute value) being less than 1, the phenotypic frequency distributions had a relatively normal distribution. This indicates that the phenotypic data was suitable for QTL mapping. For the germplasm population, the seed oil content ranged from 15.63 to 22.90% and from 14.67 to 22.63% in 2013 and 2014, respectively. The phenotypic frequency distributions of the germplasm population also had a normal distribution.

**Table 1:** Results of seed oil content in the linkage mapping and soybean germplasm populations.

Population	Parents (%)			Range (%)	Mean (%)	SD	CV (%)	Skewness	Kurtosis	Env.	*h*^2^
	M8108	MN-5	NN1138-2								
NJMN-RIL	20.33 ± 0.55	-	19.00 ± 0.44	17.20–21.37	19.69	0.95	4.82	-0.48	-0.46	2012JP	0.93
	20.57 ± 0.64	-	18.90 ± 0.26	16.60–22.03	19.64	1.14	5.8	-0.4	-0.12	2012FY	
	20.40 ± 0.26	-	19.13 ± 0.35	18.60–22.70	20.99	0.95	4.53	-0.38	-0.34	2014JP	
	19.93 ± 0.47	-	18.90 ± 0.30	17.67–23.73	20.84	1.21	5.81	-0.07	0.14	2014YC	
	21.20 ± 0.62	-	19.30 ± 0.44	18.20–22.73	20.89	0.96	4.6	-0.24	-0.42	2014HA	
F_2_	-	20.70	19.00	17.5–21.9	19.71	0.85	4.31	0.12	-0.34	2015JP	-
Germplasm	-	-	-	15.63–22.90	19.7	1.45	7.36	-0.4	-0.31	2013JP	0.88
	-	-	-	14.57–22.63	19.81	1.32	6.66	-0.48	0.23	2014JP	

In addition, the ANOVA results indicated significant differences in genotype, environment, and genotype-by-environment (Supplementary Table S1). The *h*^2^ of seed oil content in the NJMN-RIL population and germplasm population was high (0.93 and 0.88, respectively), which was consistent with previous studies (Chung et al., 2003; Panthee et al., 2005).

### Linkage Maps and QTL Mapping of Seed Oil Content in the NJMN-RIL Population

**FIGURE 1:**
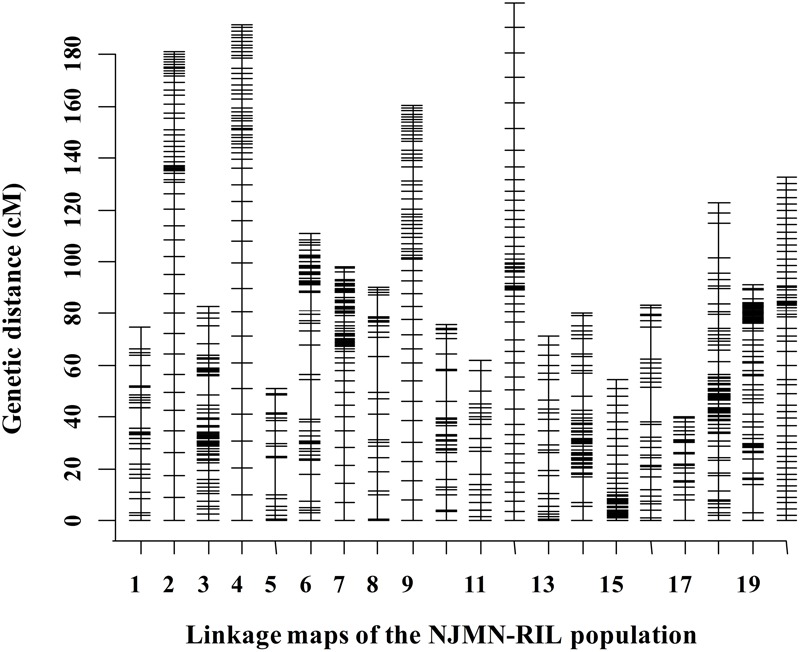
Distribution of markers in 20 linkage groups (LGs) in the NJMN-RIL population. The black bars in each LG represent mapped SLAF-seq markers. Detailed information is presented in Supplementary Table S2.

The RIL population was genotyped using SLAF-seq technology. After being filtered and quality assessed, a total of 2,086 SLAF markers were available to construct genetic maps for the NJMN-RIL population. Finally, 2,062 SLAF markers were grouped into 20 LGs. The total genetic distance of this map was 2054.50 cM. The average distance between adjacent markers was approximately 1 cM. The genetic length of 20 LGs ranged from 40.21 cM (Chr. 17) to 199.77 cM (Chr. 12). The largest LG was Chr. 18, with 338 SLAF markers. The LG with the minimum number of markers was Chr. 05, with 28 SLAF markers. The mean LG length was 102.73 cM and each chromosome contained an average of 103 markers. Detailed map information is provided in **Figure 1** and Supplementary Table S2.

**FIGURE 2:**
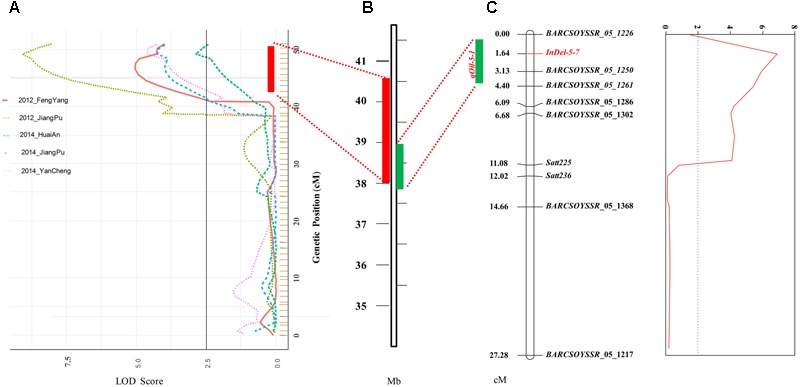
Detection and confirmation of *qOil-5-1* using *NJMN-RIL* and secondary F_2_ populations by linkage mapping. **(A)** *qOil-5-1* was detected in the NJMN-RIL population in five environments. The physical location of the confidence interval of *qOil-5-1* was approximately 2.5 Mb, from 38.04 to 40.51 Mb on chromosome 05. **(B)** Physical location of the 1- logarithm of odds (LOD) confidence interval of *qOil-5-1* on chromosome 05. **(C)** *qOil-5-1* was confirmed in the secondary F2 population. The peak marker was *InDel-5-7*. The 1- logarithm of odds (LOD) confidence interval of the QTL spanned approximately 1 Mb.

Based on the genetic map, a total of eight QTLs for seed oil content, distributed on seven chromosomes, were identified in the NJMN-RIL population across the different environments by WinQTLCart 2.5 software, with LOD scores ranging from 2.7 to 9.1 and 6.3 to 26.2% of the phenotypic variation explained by individual QTLs (**Table 2**). Among them, *qOil-5-1* was the most stable QTL detected in all environments and could explain 7.5–26.2% of the phenotypic variation. The *qOil-5-1* QTL was mapped at an approximate 6.8 cM (43.4–50.2 cM) interval in all environments (**Figure 2A** and **Table 2**). This QTL interval has a corresponding physical distance of 2.5 Mb, from 38.04 to 40.51 Mb on Chr. 05 (**Figure 2B**). The *qOil-10-1* and *qOil-14-1* QTLs could also be considered major QTLs because they were detected in four environments. The other five QTLs were detected in a single environment. In addition, the positive alleles of *qOil-3-1* and *qOil-10-1* came from *NN1138-2*, while the others came from *M8108*.

**Table 2:** Analysis of quantitative trait loci (QTLs) for seed oil content in the NJMN-RIL population.

QTLs name^a^	Chromosome	Position (cM)	LOD^b^	R^2^(%)^c^	A^d^	Confidence interval (cM)^e^	Env.	Physical region (bp)^f^
*qOil-3-1*	3	16.0	2.9	7.2	0.27	15.0–17.7	12JP	2188542–3448125
*qOil-5-1*	5	46.6	5.1	13.7	-0.43	43.4–49.2	12FY	38048316–40507761
		49.2	9.1	26.2	-0.50	46.0–50.2	12JP	
		49.2	4.2	10.6	-0.31	48.4–50.2	14JP	
		49.2	4.6	10.3	-0.40	45.8–50.2	14YC	
		49.2	2.9	7.5	-0.3	45.5–50.2	14HA	
*qOil-10-1*	10	72.2	4.9	10.7	0.43	70.6–74.2	14YC	43691698–48420826
		73.2	8.1	21.3	0.56	72.2–73.9	12FY	
		73.2	4.0	10.4	0.32	70.8–73.5	14JP	
		73.2	5.2	14.5	0.38	72.2–73.5	14HA	
*qOil-11-1*	11	48.2	2.7	6.3	-0.31	46.2–51.6	12FY	10235376–16505514
*qOil-13-1*	13	23.6	3.6	11	-0.42	23.4–27.1	14YC	23197414–28206411
*qOil-14-1*	14	37.7	5.1	12.1	-0.40	35.2–39.8	12FY	7977618–11579289
		38.7	6.3	17.4	-0.40	35.5–40.0	14JP	
		38.7	5.2	12.4	-0.43	36.1–41.4	14YC	
		37.7	5.8	16.9	-0.42	34.7–39.0	14HA	
*qOil-14-2*	14	78.6	2.7	7.6	-0.27	74.6–79.6	12JP	46374620–48292593
*qOil-17-1*	17	12.8	6.5	15.5	-0.49	10.3–13.8	14YC	361905–12470166

*^a^QTLs detected in different environments at the same, adjacent, or overlapping marker intervals were considered the same QTL. ^b^The log of odds (LOD) value at the peak likelihood of the QTL. ^c^Phenotypic variance (%) explained by the QTL. ^d^A positive value indicates that *NN1138-2* contributed the allele for an increase in seed oil content, and negative additive effects indicated that *M8108* contributed the allele for an increase in seed oil content. ^e^1-LOD support confidence intervals (confidence interval length). ^f^Physical location of the confidence interval of QTLs.*

### Confirmation of the *qOil-5-1* QTL Using a Secondary F_2_ Population

The *qOil-5-1* QTL was detected in all environments and so could be considered the most stable QTL in the NJMN-RIL population. To confirm and refine the position of this QTL, the secondary F_2_ population was used. The seed oil content ranged from 17.50 to 21.90% in this F_2_ population (**Table 1** and Supplementary Figure S1). We also constructed a genetic map with 10 polymorphic pairs for the F_2_ population (**Figure 2C**). This genetic map covered the *qOil-5-1* region (from ∼37.8 to 40.8 Mb on Chr. 05). Based on the phenotype and genetic map, a QTL was identified. The highest peak of the QTL was found at the marker *InDel-5-7* [primer sequence: F′ (5′–3′): CAAAAATACATTGAATGGAAGGCAA; R′ (5′–3′): TGACAGAGAGATATTATCCCCAGC], with an LOD score of 6.87, which strongly exceeded the threshold level. The *InDel-5-7* marker corresponded to position 38494541-38494952 bp on the reference genome (*G. max* Wm82.a1). The 1-LOD confidence interval of the QTL spanned approximately 1 Mb, from position 37.85 Mb (*BARCSOYSSR_05_1217*) to 38.90 Mb (*BARCSOYSSR_05_1261*) of the physical map, which was consistent with the RIL population mapping results (**Figures 2B,C**).

### Genome-Wide Association Study (GWAS) for Seed Oil Content

In this study, 279 soybean gremplasm lines with 59,845 (MAF > 0.05) SNPs were used to perform the association analysis. The quantile–quantile (QQ) plot showed that the MLM method was appropriate for this study and could reduce the number of false positive results (Supplementary Figure S2). Therefore, the MLM model was used for the association analysis.

**FIGURE 3:**
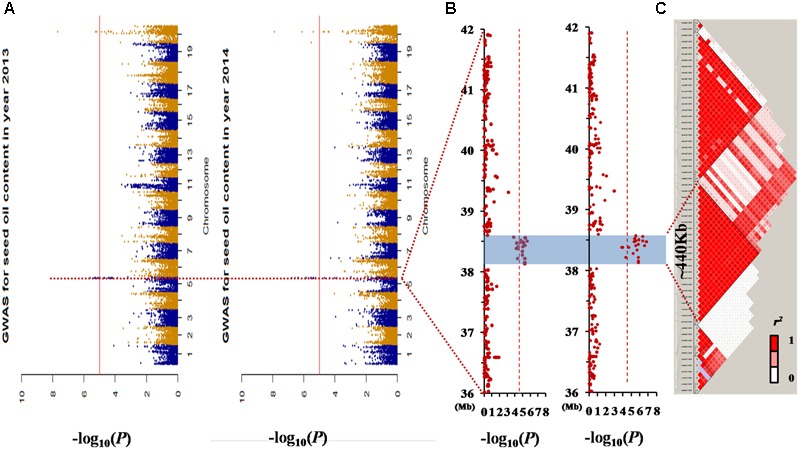
Genome-wide association study (GWAS) results for seed oil content in the germplasm population. **(A)** Manhattan plot of the seed oil content in two different environments. The dashed horizontal line depicts a significant threshold level [–*log_10_* (1/59845)]. **(B)** GWAS results of the 36–42 Mb genomic region of chromosome 05. **(C)** The distribution of linkage disequilibrium (LD) blocks of the major locus on chromosome 05. All the single nucleotide polymorphisms (SNPs) that had a significant association with seed oil content in the 2 years investigated were located in one LD block from 38.13 to 38.57 Mb.

There were two association peaks identified on Chr. 05 and on Chr. 20 in 2013; and one association peak on Chr.05 and two association peaks on Chr. 20 were identified in 2014. The phenotypic variation explained by each peak ranged from 8.5 to 12.9% (**Table 3** and **Figure 3A**).

**Table 3:** Results of a genome-wide association study (GWAS) for seed oil content.

Env.	Chromosome	Peak marker	Position (bp)	-Log_10_(*P*)^a^	*R*^2^%^b^	Association region (bp)^c^
2013JP	5	Gm05_38506373	38506373	5.8	8.8	38.03–38.99
	20	Gm20_31164168	31164168	7.4	11.8	30.68–31.64
2014JP	5	Gm05_38473956	38473956	6.8	10.8	37.99–38.95
	20	Gm20_606529	606529	5.6	8.5	0.13–1.09
	20	Gm20_31164168	31164168	8.0	12.9	30.68–31.64

*^a^*P* is the statistical *P*-value for the significance of the odds ratio in the GWAS. ^b^Phenotypic variance (%) explained by the peak marker. ^c^The region is 480 kb (LD distance) upstream and downstream of the peak marker position.*

The loci for seed oil content on Chr. 05 could be detected in both the linkage and association population. Therefore, *qOil-5-1* can be considered an important locus for seed oil content in the summer planting soybeans. To reduce the *qOil-5-1* region, we focused on the results from the association population (**Figure 3B**). The peaks on Chr. 05 were located at 38506373 and 38473956 bp in 2013 and 2014, respectively. Based on the LD distance, we extended the region of 480 kb upstream and downstream of the significantly associated SNP position. The LD was estimated from the *r*^2^ correlation between each marker in this region, with *r*^2^ values less than 0.2 considered unlinked. This region possessed two large LD blocks, one from 38.13 to 38.57 Mb and the other from 38.59 to 38.95 Mb (**Figure 3C**). However, all the SNPs showing a significant association with seed oil content in the 2 years were located within the first LD block region. This suggested that the major QTL for seed oil content was located in a small region from 38.13 to 38.57 Mb on Chr. 05.

## Discussion

### Optimizing the Population and Marker System for Mapping QTLs of Complex Traits

Quantitative trait loci mapping is an effective approach to analyze quantitative traits in plants. Linkage mapping and GWAS are commonly used to map the QTLs of complex quantitative traits. However, the linkage mapping method has a relatively low genome resolution and the GWAS results are affected by the Q and individual relationships. A combination of GWAS and linkage mapping could provide mutual authentication and enable more reliable results to be obtained. This strategy has been successfully applied in many studies (Konishi et al., 2006; Zhang D. et al., 2014; Zhang Y. et al., 2016; Zhao et al., 2015; Sun et al., 2016). Because the soybean cultivars in the Jiang-Huai River Valley usually have a relatively low seed oil content (Zhang J. W. et al., 2014), it is difficult to breed high oil content varieties by traditional breeding in this region. Molecular MAS is a possible alternative. However, little is known about the genetics of oil content in these soybean. In this study, we used 104 RILs and 279 accessions to dissect the genetic basis of seed oil content in soybean, which would provide useful information for MAS to breed high oil content varieties in this region.

The marker density should be considered during QTL mapping, regardless of which method is used. Because of the technology limitations, the genetic maps used in most of the studies mentioned above were constructed for mapping QTLs associated with seed oil content in soybean with only hundreds of restriction fragment length polymorphism, amplified fragment length polymorphism, and/or SSR markers. In addition, the interval distance between adjacent markers was large, which limits the efficiency and accuracy of QTL mapping. In our study, SLAF-seq was used to genotype a total of 104 individuals and the two parents. We constructed a genetic map with 2,062 SLAF markers for linkage mapping, and the average distance between adjacent markers was only 1 cM. For GWAS, 59,845 polymorphism SNPs with MAF >0.05 were used. Previous studies have shown that the LD of cultivated soybean is approximately 150 kb (Lam et al., 2010; Hao et al., 2012; Wen et al., 2014). Hwang et al. (2014) reported that the mean LD (*r*^2^) rapidly declined to 0.2 within 360 kb in euchromatic regions and declined to 0.2 at 9600 kb in heterochromatic regions. However, there is a high LD in the soybean genome, implying a limitation on the effectiveness of increasing marker density to improve the resolution. This genetic map and high-number of SNPs can meet the demands of linkage and a GWAS of the complex traits in soybean.

### Major QTLs for Seed Oil Content in Soybean

Soybean is a primary source of plant oil for humans (Wilcox, 2004). The breeding of high oil content varieties is an important goal of oil crop breeding programs. The QTLs controlling seed oil content have been reported many times in bi-parental populations (Mansur et al., 1996; Brummer et al., 1997; Kabelka et al., 2004; Qi et al., 2011; Wang et al., 2015). Many loci associated with soybean seed oil content have been detected by GWAS methods (Hwang et al., 2014; Bandillo et al., 2015; Wen et al., 2015). However, most QTLs are environmentally sensitive. Brummer et al. (1997) considered that in molecular-assisted breeding programs, breeders should use QTLs that are stable in a range of different environments. Thus, there is a need to identify stable QTLs that can be conveniently used for MAS and map-based cloning to determine the mechanisms of seed oil content.

In this study, the distribution of seed oil content followed a normal distribution in both the natural soybean population and in RILs, indicating that the genetic control of seed oil content is complex (Supplementary Figure S1). Although seed oil content had a high *h*^2^, the environmental instability of this trait made it difficult to detect stable QTLs in different environments. However, there were some major loci detected in different environments using one method in our study. In linkage mapping, *qOil-10-1* and *qOil-14-1* were detected in four environments, had adjacent or physically overlapping QTLs, as reported by previous studies, and might actually be the same QTL (Tajuddin et al., 2003; Wang et al., 2014). In the GWAS, the significant SNPs on Chr. 20 were found to be associated over the 2 years investigated. The peak marker, Gm20_31164168, was similar to the one identified by Vaughn et al. (2014) at 31150279 bp on Chr. 20. Therefore, our results were in good agreement with previous studies, and the QTLs identified can be treated as important targets for the identification of candidate genes involved in the modification of oil in future studies.

### Combination of Linkage and Association Mapping to Refine the Stable QTL on Chr. 05

Although the number of QTLs controlling seed oil content have been reported by linkage mapping and/or GWASs in soybean, the confirmation of such QTLs is rare. Here, we confirmed and narrowed down the interval of a stable QTL on Chr. 05 by a combination of linkage and association mapping. In the NJMN-RIL population, *qOil-5-1* was mapped at an approximate 6.8 cM interval in all environments. Based on the physical position of the SLAF markers, this genetic map interval has a corresponding physical distance of 2.5 Mb, from 38.04 to 40.51 Mb on Chr. 05, which has adjacent or physically overlapping QTLs, as reported by Mansur et al. (1996), Orf et al. (1999), and Pathan et al. (2013). In the secondary F_2_ population, the position of this QTL could be refined at a 3 cM interval between the *BARCSOYSSR_05_1226* and *BARCSOYSSR_05_1250* markers, and the peak marker corresponded to position 38494541–38494952 bp. These results indicated that the region of *qOil-5-1* is from ∼37.85 to 38.90 Mb. In the germplasm population, based on the GWAS results and the partial LD of Chr. 05, we found that all the SNPs that had a significant association with seed oil content over the 2 years investigated were located in one LD block. Taken together, this evidence strongly suggests that this stable QTL for seed oil content was located in a small region from 38.13 to 38.57 Mb on Chr. 05.

Plant oil is typically biosynthesized and accumulated in the cotyledon or endosperm tissues of seeds. The pathways for lipid biosynthesis and oil accumulation have been studied and are known to be controlled by multiple genes. Within the LD block region, there were more than 60 functional genes^2^. Thirteen of those genes were highly expressed in seed (information from Phytozome database^3^) and may be related to oil biosynthesis or oil accumulation (**Table 4**). However, the functions of these genes need to be verified in future studies. Finally, such fine-mapping of QTLs and close markers (including SNP, SSR and *InDel* markers) must be used for MAS and map-based cloning to determine the mechanisms of seed oil content.

**Table 4:** Genes within the linkage disequilibrium (LD) block that are highly expressed in soybean seed.

Gene	Start	Stop	Annotation
*Glyma05g33510*	38135689	38147080	Rhodanese-like domain-containing
*Glyma05g33540*	38166833	38170524	Ser/thr dehydratase, trp synthase
*Glyma05g33620*	38233350	38236475	Annexin
*Glyma05g33630*	38237658	38240589	Tartrate-resistant acid phosphatase type 5
*Glyma05g33770*	38328612	38331790	Sel-1-like protein, sel-1
*Glyma05g33820*	38357517	38361122	Mitochondrial ADP/ATP carrier protein
*Glyma05g33850*	38373907	38376831	Homeobox domain
*Glyma05g33930*	38429239	38433668	PCI domain
*Glyma05g33990*	38466368	38466617	
*Glyma05g34020*	38483013	38486138	
*Glyma05g34030*	38495325	38497529	Phosphatidylethanolamine-binding protein
*Glyma05g34070*	38511154	38513219	Receptor for activated protein kinase c (rack1)
*Glyma05g34090*	38526145	38528922	Bcas2 protein

## Conclusion

We identified some major QTLs for seed oil content and dissected the genetic basis of this trait in soybean using linkage and germplasm populations. A stable QTL was confined to a small interval of approximately 440 kb on Chr. 05 by combining a linkage and genome-wide association mapping approach. A genetic map was constructed using more than 2,062 SLAF markers for the NJMN-RIL population in this study, which can provide a good foundation for analyzing quantitative traits. The results of this study are important for future map-based gene cloning and also provide support for the implementation of MAS for breeding soybean with a high seed oil content in the Jiang-Huai River Valley.

## Author Contributions

TZ conceived the research; TZ and JG designed the research; YC, SL, ZW, FC, and JK performed the experiments and analyzed the data. YC drafted the manuscript. TZ revised the paper.

## Conflict of Interest Statement

The authors declare that the research was conducted in the absence of any commercial or financial relationships that could be construed as a potential conflict of interest.

## Footnotes

1 http://www.soybase.org/

2 http://www.soybase.org

3 http://phytozome.net
