# Supplementary material for: Identification of Major Quantitative Trait Loci for Seed Oil Content in Soybeans by Combining Linkage and Genome-Wide Association Mapping
Source: Front Plant Sci. 2017 Jul 12;8:1222. doi: 10.3389/fpls.2017.01222 (PMC5506190; doi:10.3389/fpls.2017.01222)
Supplement: Supplementary file 1 [file Table_1.docx]

*Supplementary Material*

**Identification of Major Quantitative Trait Loci for Seed Oil Content in Soybeans by Combining Linkage and Genome-wide Association Mapping**

**First Author*：Yongce Cao**

***Corresponding author**

Tuanjie Zhao

[tjzhao@njau.edu.cn](mailto:tjzhao@njau.edu.cn)

**Supplementary materials**

**Table S1** Analysis of variance (ANOVA) of seed oil content in the NJMN-RIL and germplasm populations

| population | Source | *DF*^a^ | *MS*^b^ | *F*^c^ |
| --- | --- | --- | --- | --- |
| NJMN-RIL | Genotype | 103 | 12.26 | 39.76^****^ |
|  | Block within Environment | 10 | 5.80 | 18.8^****^ |
|  | Environment | 4 | 136.44 | 442.61^****^ |
|  | Genotype × environment | 410 | 0.87 | 2.81^***^ |
|  | Error | 978 | 0.31 |  |
|  |  |  |  |  |
| Germplasm | Genotype | 272 | 10.06 | 16.55^****^ |
|  | Block within Environment | 4 | 1.49 | 2.46^*^ |
|  | Environment | 1 | 8.86 | 14.58^****^ |
|  | Genotype × environment | 271 | 1.25 | 2.05^****^ |
|  | Error | 1036 | 0.61 |  |

^*^*P* <0.05 ^****^*P* <0.0001

^a^ Degrees of freedom

^b^ Mean square

^c^ F value is used to determine significance

**Table S2** Summary of the distribution of mapped markers in a genetic linkage map with the NJMN-RIL mapping population

| Chromosome | Marker numbers | Linkage distance (cM) | Average interval distance (cM) |
| --- | --- | --- | --- |
| Chr. 01 | 88 | 74.49 | 0.85 |
| Chr. 02 | 56 | 180.97 | 3.23 |
| Chr. 03 | 153 | 82.79 | 0.54 |
| Chr. 04 | 52 | 191.54 | 3.68 |
| Chr. 05 | 28 | 50.97 | 1.82 |
| Chr. 06 | 231 | 111.08 | 0.48 |
| Chr. 07 | 123 | 98.29 | 0.80 |
| Chr. 08 | 55 | 90.13 | 1.64 |
| Chr. 09 | 79 | 160.47 | 2.03 |
| Chr. 10 | 152 | 75.51 | 0.50 |
| Chr. 11 | 51 | 61.90 | 1.21 |
| Chr. 12 | 81 | 199.77 | 2.47 |
| Chr. 13 | 54 | 71.24 | 1.32 |
| Chr. 14 | 91 | 80.04 | 0.88 |
| Chr. 15 | 83 | 54.32 | 0.65 |
| Chr. 16 | 41 | 83.31 | 2.03 |
| Chr. 17 | 53 | 40.21 | 0.76 |
| Chr. 18 | 273 | 123.06 | 0.45 |
| Chr. 19 | 228 | 91.51 | 0.40 |
| Chr. 20 | 90 | 132.90 | 1.48 |
| total | 2062 | 2054.50 | 1.00 |


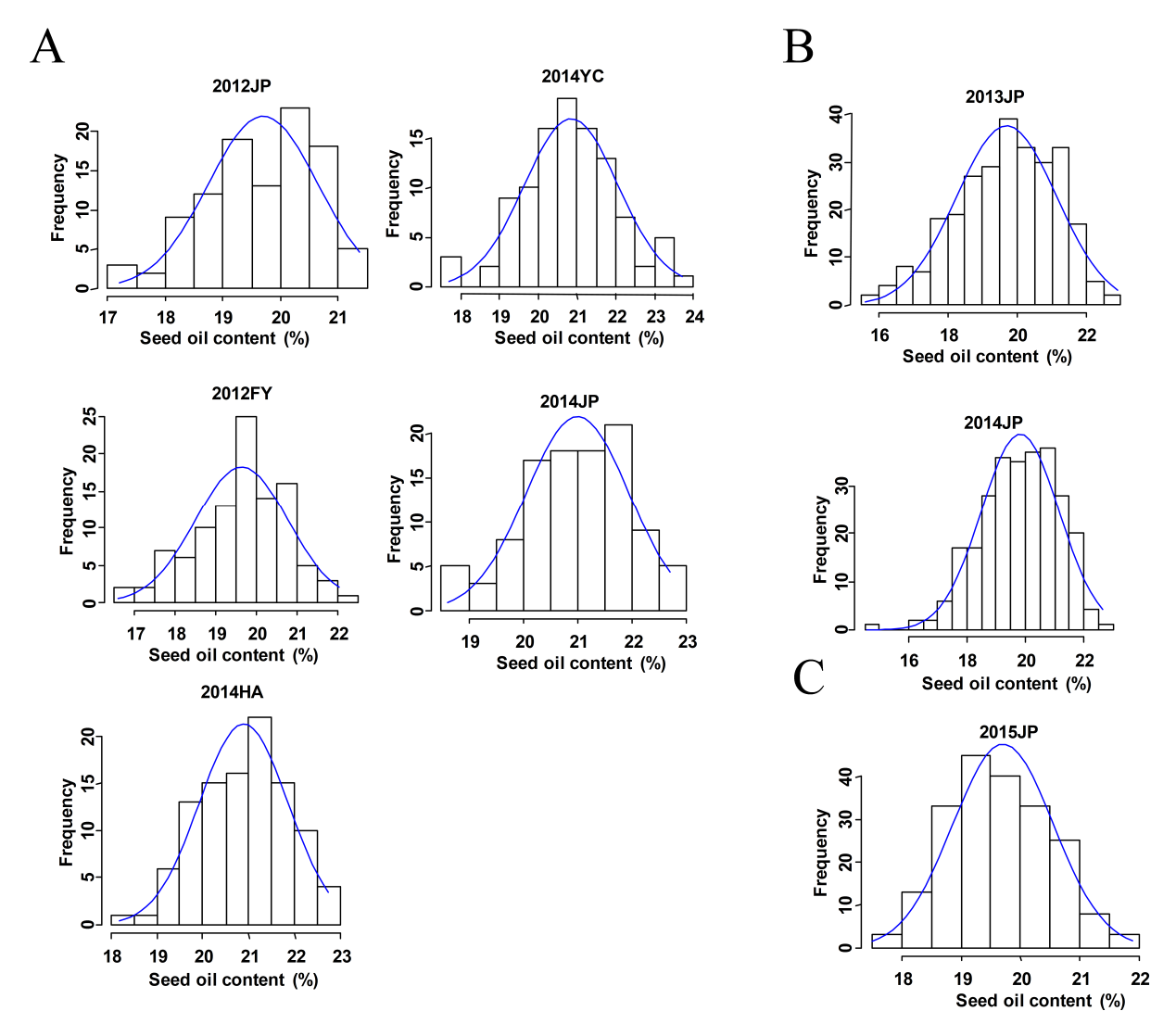


**Figure S1** Frequency distribution of seed oil content in the linkage mapping and germplasm populations. (A) Seed oil content in the NJMN-RIL population in five different environments. (B) Seed oil content in the germplasm population in two different environments. (C) Seed oil content in the F_2_ population.


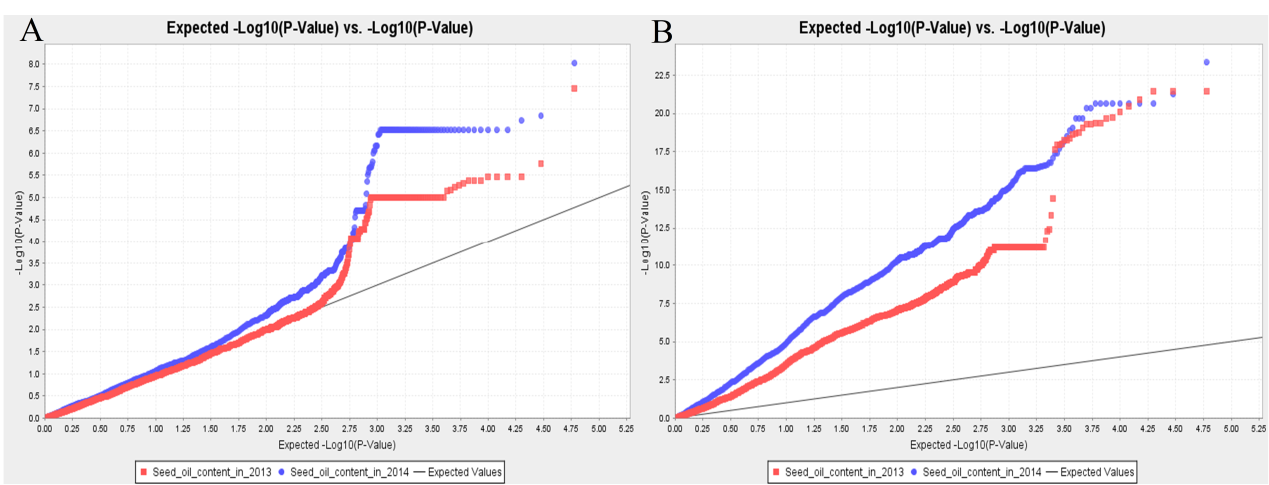


**Figure S2** Quantile–quantile (Q-Q) plots of estimated −log_10_ (*P*). (A) Q-Q plot of the mixed linear model (MLM) model. (B) Q-Q plot of the generalized linear model (GLM) model.
